# Towards conductive textiles: coating polymeric fibres with graphene

**DOI:** 10.1038/s41598-017-04453-7

**Published:** 2017-06-26

**Authors:** Ana I. S. Neves, Daniela P. Rodrigues, Adolfo De Sanctis, Elias Torres Alonso, Maria S. Pereira, Vitor S. Amaral, Luis V. Melo, Saverio Russo, Isabel de Schrijver, Helena Alves, Monica F. Craciun

**Affiliations:** 1 Centre for Graphene Science, College of Engineering, Mathematics and Physical Sciences, University of Exeter, EX4 4QF Exeter, United Kingdom; 2 INESC-MN and IN, Rua Alves Redol 9, 1000-029 Lisbon, Portugal; 3 Department of Physics and CICECO, University of Aveiro, 3810-130 Aveiro, Portugal; 4 Department of Physics, Instituto Superior Técnico, University of Lisbon, Av. Rovisco Pais 1, 1040-001 Lisbon, Portugal; 5 CenTexBel, Technologiepark-Zwijnaarde 7, 9052 Ghent, Belgium

## Abstract

Conducting fibres are essential to the development of e-textiles. We demonstrate a method to make common insulating textile fibres conductive, by coating them with graphene. The resulting fibres display sheet resistance values as low as 600 Ωsq^−1^, demonstrating that the high conductivity of graphene is not lost when transferred to textile fibres. An extensive microscopic study of the surface of graphene-coated fibres is presented. We show that this method can be employed to textile fibres of different materials, sizes and shapes, and to different types of graphene. These graphene-based conductive fibres can be used as a platform to build integrated electronic devices directly in textiles.

## Introduction

The development of devices on textiles such as sensors^1^, photodetectors^2^, transistors^3^, electro-luminescent devices^4^, supercapacitors^5^ and solar cells^6^ is attracting great interest and has led to the emergence of the field of smart textiles. Smart textiles can find an enormous range of applications in several fields, including healthcare, military and fashion^7^. Since the concept of textiles is much wider than clothes and garments, the applications can extend to aviation, automotive and transport, construction, geo-textiles and packaging. While most commercial applications of smart textiles rely on conventional hardware simply mounted onto textiles, the integration of specific functionalities directly on textile fibres promises to revolutionize the field of wearable electronics. With the recent advances in nanotechnology and materials engineering, different functionalities can now be incorporated into textile fibres, such as antibacterial properties, static elimination, and electric conductivity^8^.

Conducting fibres are an important component of any e-textile, not only because they can be used as lightweight wiring for simple textile-based electronic components, but also because they can provide a platform for building electronic devices directly on textile fibres. For instance, such fibres can be used as gate electrodes for field-effect transistors, or bottom electrodes for light-emitting diodes and photovoltaic devices. Conductive textile fibres are currently used as a stronger and more flexible weight-saving material in the aviation sector, where the aircraft weight and fuel consumption are reduced by replacing metal wiring with electrically conductive cotton fibres like ARACON®. The most common approach to produce conductive fibres consists in mixing an insulating polymeric matrix with a conductive component, which can be a conducting polymer such as PEDOT:PSS^9^, silver nanowires^10^, nanocarbon fillers^11^, or their hybrids^12–15^. These composite fibres are usually produced by wet-spinning of the polymer with a suspension of the active conducting material, or via electrospinning^16^, techniques that require a large consumption of expensive materials and that can cause the loss of flexibility and transparency. A different approach relies on polymer-free fabrication of conducting fibres, but the methods include the use of strong acids and coagulants^17^, which greatly limits the potential for scaling up and commercialization. Another strategy is to impregnate fibres with conductive materials after they have been manufactured. This method has been used for fibres, yarns and fabrics that are highly porous with a complex structure of microfibrils, such as cellulosic fibres. These inks and dyes make use of several types of conductive materials, such as aluminium^18^, carbon nanotubes^19^, and graphene^20^, and have been in the base of demonstrations of wearable and stretchable electronics, including integration onto surfaces of live plants and insects^21, 22^. One of the limitations of this methodology is that such multifilament fibres and fabrics, compared to fibres coated prior or during manufacturing, tend to lose the conductive filling more easily if not completely encapsulated, and can pose end-of-life environmental concerns.

A more practicable emerging strategy to prepare conductive textile fibres is to coat insulating fibres with conductive atomically thin two dimensional layers such as graphene^23^. Graphene, a monoatomic carbon layer, is the strongest known material, the best electrical and thermal conductor which is also mechanically flexible and transparent^24^. Thus, it represents a radical alternative to conventional technologies as it can bend, stretch, compress, twist and deform into complex shapes while maintaining the same levels of performance and reliability^25^. There are already several examples of graphene-based textiles with different functionalities and for different applications^26^. The coating we propose is performed by electrostatic adhesion of graphene at the surface of monofilament fibres and does not involve impregnating an agglomerate of fibres. The adhesion of the graphene coating to the textile fibres is strong and durable, and a straightforward passivation can be achieved by encapsulation with an insulating polymeric layer. This method was developed for tape-shaped polypropylene (PP) and bio-based polylactic acid (PLA) fibres, two polymers with widespread use in the textile industry^27^. This approach depends on the size of the graphene sample, usually in the centimetre range, and although this might not be suitable for electronic wiring, it is appropriate to build electronic devices directly on textile fibres.

In this work, we demonstrate that graphene can be transferred to a large variety of thermoplastic monofilament textile fibres of different types and shapes. To further advance the development of this technique, it is important to understand the various factors that can influence the conductivity achieved by coating textile fibres with graphene. Surface topography and chemical nature seem to be determinant in the conductivity achieved. On the other hand, cracks and tears in the graphene coating will result in a decrease in conductivity, and therefore it is important to establish their origin. Thus, our present study aims at: (1) establishing how the above-mentioned factors actually influence the quality of the graphene coverage and how it correlates with the resulting conductivity; and (2) explore the suitability of our coating method for different materials, sizes and shapes.

## Results

Slight modifications in the fabrication of the fibres can result in significant changes in the fibre surface. To evaluate the influence of fibre topography in the graphene coating processed, we present a comparative study using PP fibres manufactured with different extrusion parameters. Different sources of polymer can also determine the topography and influence chemical nature of the surface, changing the quality of the graphene coating. To address this, a comparison between two formulations of PLA-based fibres was performed. Furthermore, the impact of ultraviolet-ozone (UVO) treatment on the surface of the fibres is discussed. Finally, we have employed the same methodology to different materials, such as polyethylene (PE) and nylon, and to different fibre size and shape, cylindrical (nylon) and tape-shaped (PP, PE, PLA).

**Figure 1:**
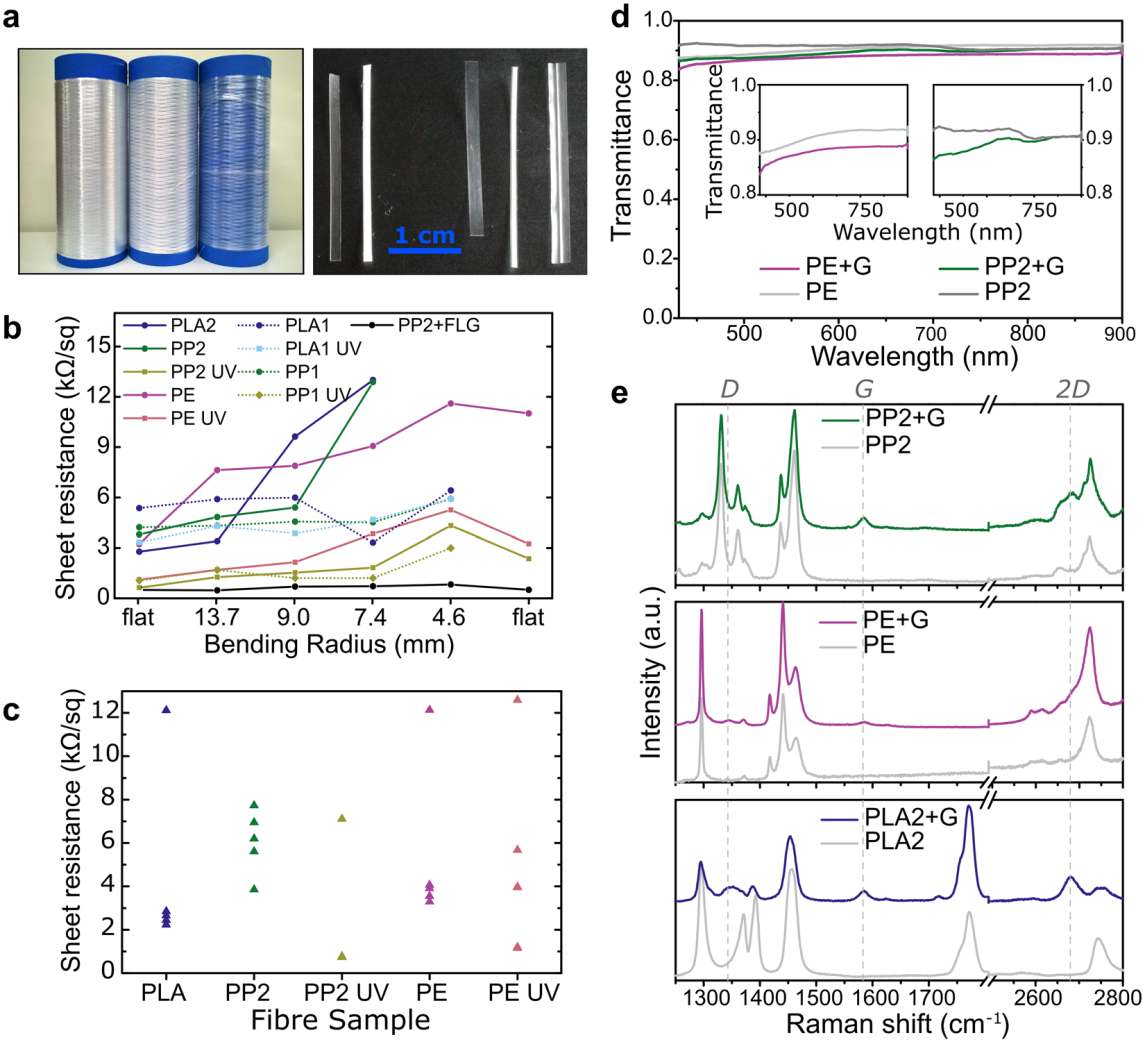
(**a**) Photo of the fibres in reels, left to right: PP2, PE, PLA2 (left) and of the fibres cut to *ca*. 3 cm length, left to right: PP1, PLA1, PP2, PLA2, PE (right). (**b**) Sheet resistance as a function of bending radius for samples of each coated fibre. In-plane measurements were performed before and after the bending cycle (“flat” in the x-axis). (**c**) Distribution of sheet resistance values for several coated samples of each fibre. (**d**) Transmittance as a function of wavelength for bare PP2 and PE, and graphene-coated (+G) PP2 and PE fibres. Insets show detail of transmittance for PP2 and PE. (**e**) Raman spectra of the bare fibres and graphene-coated (+G) fibres. Vertical dashed lines at 1345, 1685 and 2685 cm^−1^ correspond to the D, G and 2D peak positions, respectively.

Figure 1a shows photos of the textile fibres used in this work as reels (left) and cut to size (right). The fibres were coated with monolayer graphene following a method described previously^23^, which consists in etching the metal substrate in which the graphene was grown by CVD (Chemical Vapour Deposition), Cu in the case of monolayer graphene^28^ and Ni for few-layer graphene (FLG), and transferring the supported graphene onto the textile fibres immobilized on a rigid substrate. To evaluate the effect of fibre topography and different materials, extrusion parameters and precursor materials were varied. The difference between fibres PP1 and PP2 is only due to different extrusion parameters in their production, whereas PLA1 and PLA2 were extruded from different grade precursors. The difference in polymeric material used to prepare PLA1 and PLA2 is demonstrated by the Raman spectra of the two PLA fibres from both formulations, with PLA1 displaying two additional bands at 1615 and 1717 cm^−1^ compared to PLA2 (see Supplementary Fig. S1). As for both PP-based fibres, there is a good superposition between PP1 and PP2 Raman spectra, apart from minor differences in peak intensity, evidencing that both fibres are made from the same polymer.

To assess the electrical conductivity of the fibres we measured the sheet resistance for a variety of conditions. Figure 1b shows the sheet resistance values for different types of fibres coated with graphene, untreated (lines and circles) and previously submitted to UVO treatment (lines and squares), as well as their behaviour upon bending, starting in a flat position, then down to a radius of 4.6 mm, and back to a flat position. This study was performed to assess if the failure observed was due to the fibre itself failing or from damage to the graphene coating, and does not intend to be a systematic study. In general, the sheet resistance of the fibres coated with graphene is lower for fibres produced to have an improved surface (*i.e*. PP2, PLA2 and PE, represented in full lines in Fig. 1b), all falling below *ca*. 4 kΩsq^−1^, with UVO treatment bringing values even lower than 1 kΩsq^−1^. However, there seems to be a more pronounced increase in sheet resistance upon bending as compared to the PP1 and PLA1 fibres (PP1 and PLA1, dotted lines in Fig. 1b). This may be because both PP2 and PLA2 fibres are thinner and more fragile compared with PP1 and PLA1. The effect of UVO treatment in increasing the conductivity of the graphene-coated fibre samples is evident, but we found that there is a larger dispersion in sheet resistance values as the number of samples increases (Fig. 1c). This apparent irreproducibility of the UVO effect on sheet resistance is related to the fact that this treatment visibly contributes to the degradation of the fibres to some extent, particularly in the case of the more fragile and biodegradable PLA-based fibres. On the other hand, very often the sheet resistance of a fibre decreases around the bending radii of 9.0 and 7.4 mm. Although the fibres are being subjected to strain in these bending measurements, and sometimes fail completely, they are also recovering to their natural position, since they are kept in reels after the production, and therefore they have a natural curvature to them.

Transmittance measurements were performed for both types of transparent fibres, PP and PE, to evaluate the effect of graphene coating on their transparency, and are shown in Fig. 1d. A loss of only 2% and 3% in mean transmittance was observed for graphene-coated PP2 (Fig. 1d, in pink, right inset) and PE (Fig. 1d, in green, left inset), respectively, over the wavelength range between 430–900 nm, which covers the visible and near infra-red portions of the spectrum. These values are in accordance with a 2.3% absorbance for monolayer graphene^25^. The Raman spectra of fibres of the three materials (PP2, PE and PLA2), before and after the graphene transfer, are shown in Fig. 1e. For all graphene-coated fibres it was possible to identify the graphene G band at 1585 cm^−1^. The 2D peak was clearly visible for graphene-coated PP1 and PLA2, at 2685 cm^−1^, as well as a small D peak for PE and PLA2. These values match those found for G and 2D bands of the same type of graphene transferred to SiO_2_ (see Supplementary Fig. S2; more extended Raman spectra of the graphene-coated fibres is shown in Supplementary Fig. S3, along with details of the G and 2D peaks and corresponding integrations for PP2, PE and PLA2).

**Figure 2:**
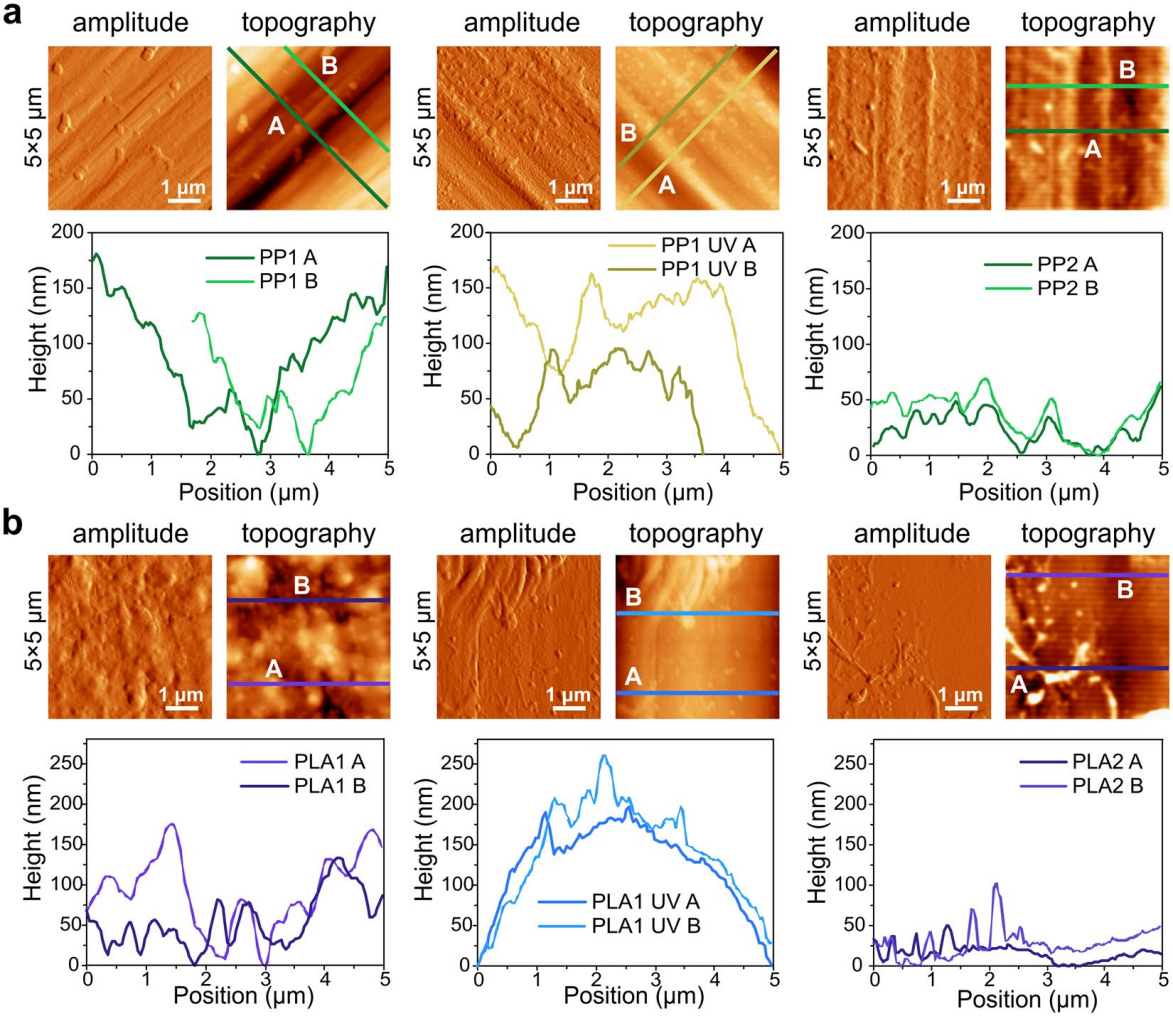
AFM amplitude and topography 5 × 5 μm images of (**a**) PP fibres before coating: PP1 (left); UVO-treated PP1 (middle); PP2 (right) and corresponding height profiles of transverse lines with respect to the extrusion axis (bottom); and (**b**) PLA fibres before coating: PLA1 (left); UVO-treated PLA1 (middle); PLA2 (right) and corresponding height profiles of transverse lines with respect to the extrusion axis (bottom). All height profiles were taken at 45° with respect to the extrusion axis.

To study the factors that lead to the observed differences in sheet resistance, it is important to understand the influence of the topography of the fibres on the effectiveness of the graphene coating. A non-contact optical method was used to determine the macroscopic surface parameters of the untreated fibres. The images obtained are shown in Supplementary Figs S4, S5 and S6, and the parameters are listed in Supplementary Table S1. Figure 2 shows the Atomic Force Microscopy (AFM) amplitude and topography for 5 × 5μm images of the PP fibres before coating and corresponding height profiles taken at the highlighted lines parallel to the extrusion axis. Compared to PP1, PP2 fibres show considerable differences in terms of AFM topography. PP2 does indeed have a smoother height profile (Fig. 2a, right) with less pronounced height differences than PP1 (Fig. 2a, left). The UVO treatment created a fine roughness throughout the whole surface, which seems to create more points where the graphene sheet can effectively adhere to the surface of the fibre (Fig. 2a, middle). The same conclusions are valid for larger and smaller AFM scanning areas (30 × 30 μm and 1 × 1 μm images, see Supplementary Fig. S7). Although in terms of overall thickness and surface features, PP1 and PP2 are very similar (see Supplementary Table S1), in a smaller scale AFM shows that PP2 has areas with less pronounced features than PP1, which is also in accordance with the smaller Kurtosis value in PP2. A similar study was performed for the PLA-based fibres PLA1 and PLA2 (Fig. 2b and Supplementary Fig. S8), showing that the changes in polymer source grade do not have a substantial impact on the surface morphology in the AFM scale. However, the difference in roughness is much more significant macroscopically, with PLA2 is rougher than PLA1, showing visible ridges perpendicular to the extrusion lines (Supplementary Fig. S5). On the other hand, we found that the UVO treatment does change the surface of PLA-fibres significantly (Fig. 2b, middle). All three monofilament tape-shaped samples, PP2, PLA2 and PE were also subjected to UVO treatment. Both PLA1 and PLA2 fibres often appeared to be damaged after UVO-treatment, particularly towards the edges, where propagating cracks and microfibrils tearing from the sample were clearly visible to the naked eye (see Supplementary Fig. S8, top middle, where a loose microfibril is clearly noticeable). The damage induced by the UVO treatment is even more severe in PLA2 fibres. The protuberances that are observed on the fibres as a consequence of the UVO treatment are probably a sign of that degradation, and biodegradable polymers like those based on PLA are prone to be damaged even more rapidly. These protuberances can be thermally caused or appear due to the chemical reactions of oxygen radicals at the surface of the polymer. Attempted coating of these UVO-treated PLA2 fibres with graphene was unsuccessful, as all the samples remained insulating after the graphene transfer. The immersion in warm acetone during the PMMA ((poly(methyl methacrylate)) cleaning step seemed to damage the fibres even further, causing them to curl and shrink. It should be noted that the UVO treatment was performed under the same conditions for all fibres, and even though the bio-based PLA1 fibres also showed some signs of quicker degradation when subjected to the UVO treatment, it was still possible to achieve considerably low sheet resistance. The fact that PLA2 fibres are slightly smaller than PLA1, 1.0 mm wide and 0.07 mm thick, compared to 1.2 mm wide and 0.1 mm thick, may also account for the fact that the formers are more fragile and easily degradable.

**Figure 3:**
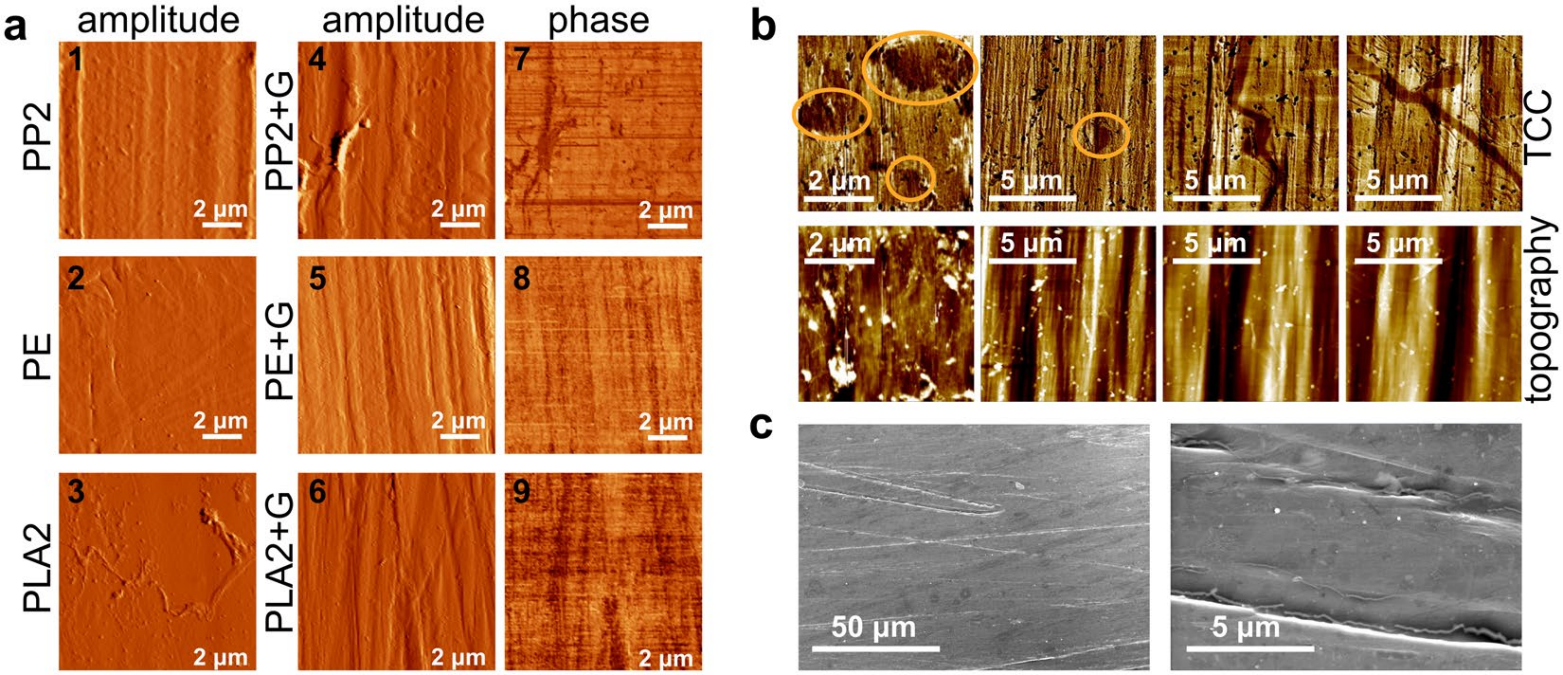
(**a**) Atomic Force Microscopy amplitude images of the fibres before coating: PP1 (1); PE (2) and PLA (3); amplitude and phase images of graphene-coated fibres: PP (4 and 7); PE (5 and 8) and PLA (6 and 9) showing a continuous graphene coverage. (**b**) Scanning Thermal Microscopy thermal conductivity contrast (TCC, top row) and topography (bottom row) images. (**c**) Scanning Electron Microscopy images showing details of holes and cracks in the graphene coverage of PP textile fibres.

An AFM study of the graphene-coated fibres also provides relevant information concerning the quality of graphene coverage at the surface. In particular, given that AFM phase imaging is capable of sensing variations in stiffness, we can distinguish between parts of the surface of the fibre that are exposed from those that are fully covered with graphene. Figure 3a shows the AFM amplitude images of the fibres before and after coating, as well as the AFM phase images of the graphene-coated fibres. All AFM phase images show a uniform graphene coverage of samples coated with no pre-treatment (Fig. 3a7, 8 and 9). These results demonstrate that a continuous surface coverage is also achievable without UVO treatment. Moreover, a detailed analysis of the topography of the graphene-coated fibres does not support the hypothesis that the tears and cracks in the graphene layer covering the surface are caused by significant differences in height. In fact, they show that several samples of PP1 and PLA1 displayed large differences in phase AFM, which correspond to tears and cracks, in areas where the topography is rather smooth (Supplementary Fig. S9, top left and middle), and areas with large differences in height that do not coincide with the discontinuities revealed by the phase images (Supplementary Fig. S9, bottom left and middle). Additionally, PP2 and PLA2 show continuous graphene coverage in areas where topography shows abrupt variations (see Supplementary Fig. S9 top and bottom right). Nonetheless, whereas continuous graphene coverage was only observed for UVO-treated PLA1 fibres, for PLA2 no pre-treatment was needed, and that might be due to the differences between both PLA sources used in the production of the fibres and how they influence the chemistry at the surface. These differences can play an important role during the graphene transfer, namely in terms of wettability, since the transfer is performed in an aqueous medium. There are essentially two ways of improving wettability in polymers: by changing surface energy and/or topography^29^. It is important to note that the UVO treatment not only changes the topography of the textile fibres, but it also causes the chemistry of the surface of the fibre to change. We observed that the wettability of all polymers improved after UVO treatment, resulting in a decrease in repulsion of the water droplets that separate the floating PMMA-supported graphene sheet, and the surface of the fibre during the transfer process, facilitating adhesion. Common methods of improving wettability, and consequently dyeability, of polymers include cold oxygen-based plasma treatment and the use of siloxane-bearing coatings^30^. As well as UVO, oxygen plasma treatment of polymers also induces ageing and degradation, namely due to formation of free radicals at the surface, which in turn can create a cross-linked polymeric layer. This layer is both rougher and has significantly different surface energy than that of the untreated polymer, and that is accountable for the increase in wettability, much like what happens during the UVO treatment. Nevertheless, UVO treatment is gentler than plasma treatment, as it has been documented in the literature, mainly as methods commonly used to clean organic residues on silicon-based surfaces^31^.

Besides AFM phase imaging, Scanning Thermal Microscopy (SThM) can also be used to assess the extent and quality of graphene coverage at the surface of the textile fibres. Surface topography and thermal conductivity contrast (TCC) often show a direct correlation in SThM. Surface roughness induces changes in the tip-sample contact area which alters the head flux between tip and sample. Abrupt depressions (and elevations) in the sample surface will translate in an apparent increase (and decrease) in thermal conductivity contrast. Although these textile fibres have been designed and produced as to have smoother surfaces, they are still inherently rough due to the extrusion process in which they were manufactured. This means that, for most samples, particularly with the PLA and PE-based textile fibres, TCC is mostly a feature of topography and actually not related with changes in the thermal conductivity of the surface. This is the reason why the SThM study is limited to PP fibres, as this new set of PP2 fibres displayed visibly smoother surfaces than all the other fibres used in this work. We have indeed found some cracks and holes in some graphene-coated PP samples which are undoubtedly non topography-induced (Fig. 3b). The same type of cracks was also visible in SEM (Scanning Electron Microscopy) images, as depicted in Fig. 3c. A comparative optical microscopy study on the effect of bending on the surface of the sample, subjecting coated PP fibres to severe and moderate bending, did not show a direct correlation between the bending stress and the appearance of cracks. For this reason, we believe that these cracks are not due to the handling and bending of the samples after the graphene transfer, but due to the manipulation of graphene sheets during the transfer process itself. The actual stress to which graphene is subjected during the wet transfer procedure, particularly as the floating PMMA-supported graphene sheet is lifted with the target sample, is most probably what is causing these cracks. Using a thicker layer of supporting PMMA would minimize the stress applied to the graphene sheet, with the drawback of generating yet more PMMA residue at the surface of the coated fibres. In fact, these PMMA residues are often visible in our studies, both in the AFM and SEM imaging (see Supplementary Fig. S10).

**Figure 4:**
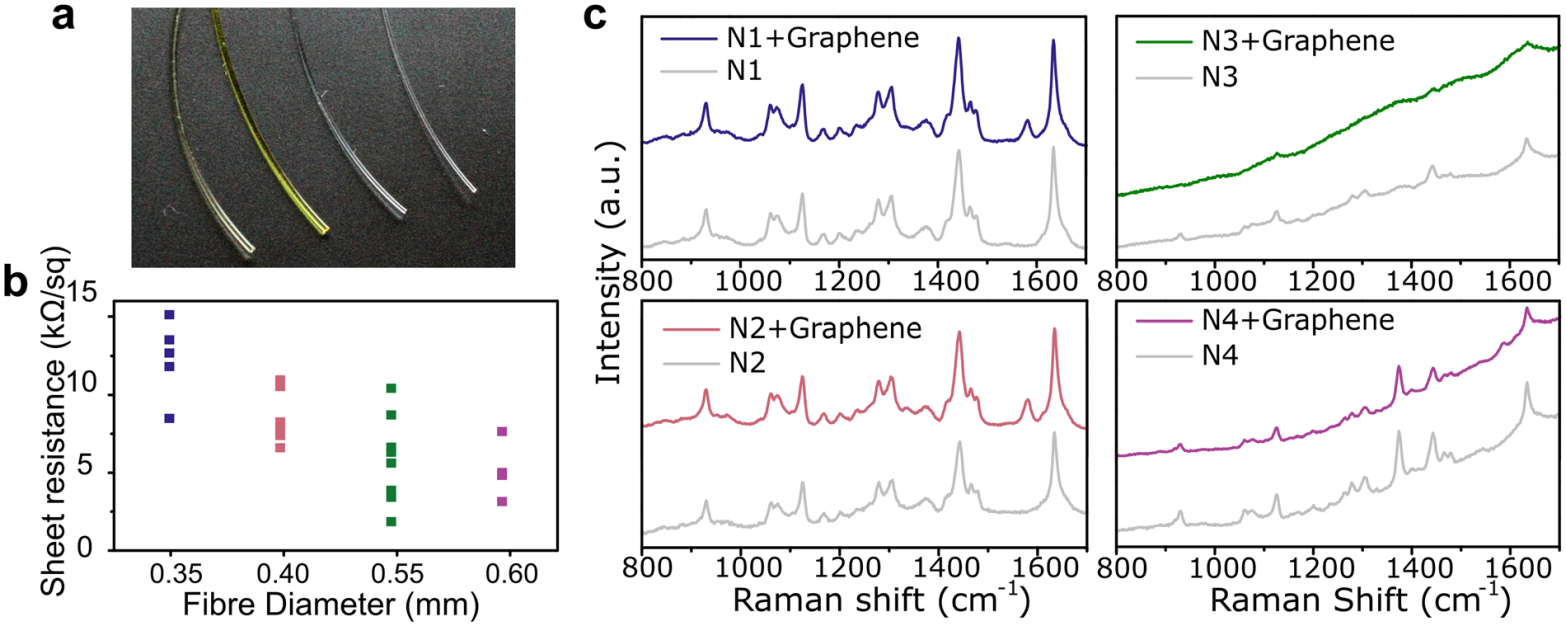
(**a**) Photo of the 4 samples, left to right: N1, N2, N4 and N4. (**b**) Sheet resistance for graphene-coated nylon fibres N1 (blue); N2 (pink); N3 (green) and N4 (purple). (**c**) Raman spectra of bare fibres (grey) and corresponding graphene-coated fibres (same colour scheme as in (**a**)).

To demonstrate the flexibility of the methodology we have developed to coat textile fibres with graphene, we performed a similar study on cylindrical shaped fibres using commercially available nylon fishing lines. As mentioned above, these fibres pose another challenge, they are cylindrical and considerably smaller than the tape-shaped fibres, with diameters ranging from 0.35 to 0.6 mm (Fig. 4a). We have assumed a graphene coverage of half a cylinder, which we believe is the maximum coverage that can be achieved with this method in cylindrical samples. This means that the sheet resistance is most likely underestimated, since the values are normalized using the aspect ratio (width/length) of the graphene sheet that is actually covering the sample, and this area is likely to be smaller than a full half cylinder coverage. Figure 4b shows the dispersion of sheet resistance of the graphene-coated nylon fibres, with values between *ca*. 2 and 15 kΩsq^−1^, which is of the same order of magnitude as the sheet resistance of the tape-shaped fibres. The conductivity of the nylon fibres increases with diameter because this increases the aspect ratio of the graphene-covered surface, which is in line with what we observed for the tape shaped fibres as well. For larger areas the electro-static adhesion of graphene is facilitated and furthermore, the effect of eventual cracks and discontinuities in the coating is minimized, which contributes to increased electrical conductivity. In any case, we found continuous graphene coverage for all samples (AFM images of N1 and N4 depicted in Supplementary Fig. S11), with both amplitude and phase AFM images of the coated fibres showing only a few drop-shaped contaminations which are probably due to PMMA residue, particularly visible in the case of P1 (see Supplementary Fig. S9c and e). As with the tape-shaped fibres, the G peak region of the Raman spectra was used to identify the presence of graphene at the surface of the nylon fibres, as show in Fig. 4c. The G peak is clearly visible in the Raman spectra of N1 and N2 (top and bottom left in Fig. 4c) at *ca*. 1580 cm^−1^. Fibres N3 and N4 are dyed (third and fourth fibres from the right in Fig. 4a, dyed in yellow and green) and exhibit a significant degree of fluorescence in the Raman spectra, as can be seen on the right-hand side of Fig. 4c. A small G peak is still visible in the Raman spectra of N3 (top right in Fig. 4c), but slightly shifted at 1586 cm^−1^, indicating doping of graphene. This doping effect may be due to the dye, and may also be accountable for the lower sheet resistance measured in both N3 and N4 samples. This will be addressed in further studies.

The potential of our method to cover textile fibres with a conductive graphene coating was further demonstrated by using a different type of graphene. Whereas all previous samples were coated with monolayer graphene, we have successfully coated PP2 fibres with few-layer graphene (FLG). The Raman spectrum of FLG-coated PP2 fibres is shown in Supplementary Fig. S12. These samples show 77% transparency, accounting for the increased number of graphene layers, as shown in Supplementary Fig. S13. Sheet resistance behaviour upon bending is shown in Fig. 1b (in black), with values lower than the most conducting fibres coated with monolayer graphene. Thus, using FLG we have achieved resistance values as low as 600 Ωsq^−1^, which is desirable for wiring textile-based electronic components. Furthermore, the increase in sheet resistance upon bending is even less significant with FLG-coated PP2 than with fibres coated with monolayer graphene, with a full recovery of the resistance after bending. This may be due to the fact that, even if the upper graphene layers are damaged with the bending stress, the remaining layers will still be continuous. AFM imaging of FLG-coated PP2 samples is presented in Supplementary Fig. S14, showing two different FLG-coated samples. One of the samples (a and b) shows what seem to be portions of the upper graphene sheets that are cracked and lifted up towards the edges, whereas the other sample (c and d) seems to have a much smoother and homogeneous surface. Both samples have similar values of sheet resistance, *ca*. 600 Ωsq^−1^. These values are slightly higher than those reported in the literature, of the order of a few hundreds of Ωsq^−1^ ^32–34^, and those of the same type of FLG transferred onto glass, 200–300 Ωsq^−1^.

**Figure 5:**
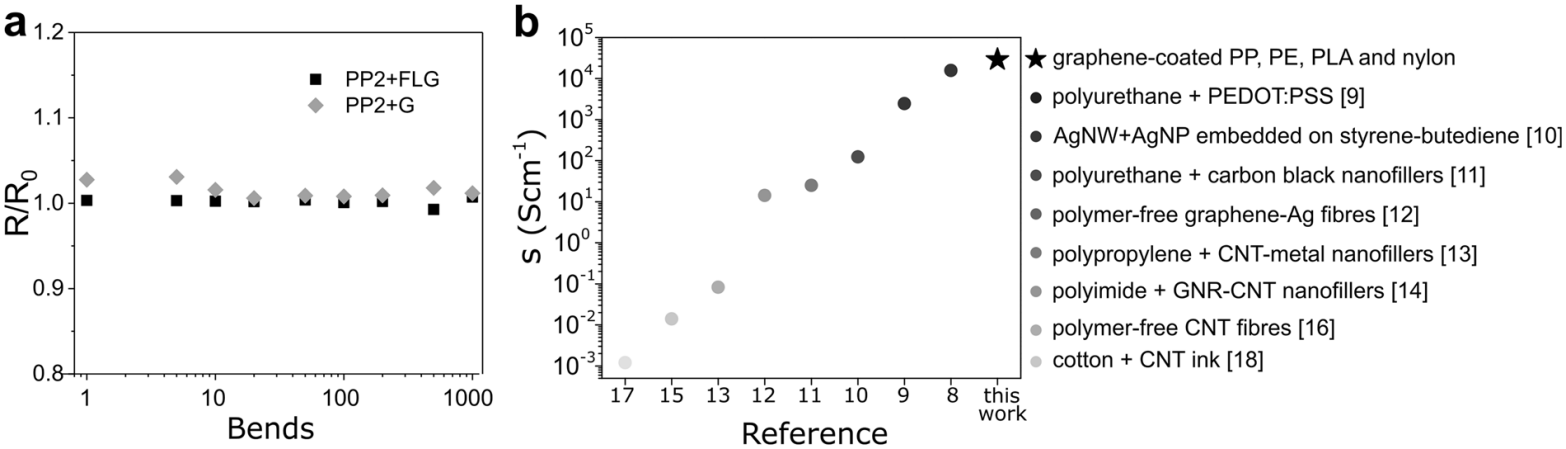
(**a**) Change in resistance upon 1000 bending cycles with bending radius of 5 mm for PP2+G (grey diamonds) and PP2 + FLG (black squares). (**b**) Comparison between the conductivity of textile fibres presented in this work and in the literature, taking into account the thickness of the conductive layer.

To demonstrate the mechanical resilience of our samples, and to directly compare the effect of bending on monolayer of few-layer graphene coating, we monitored the resistance of PP2 fibres while subjected to 1000 bending cycles, with a fixed bending radius of 5 mm. Figure 5a shows the change in sheet resistance, normalized to that of the device before bending for PP2 coated with monolayer graphene (dark yellow) and FLG (black). These measurements demonstrated that the resistance of either fibres does not change significantly during a large number of bending cycles.

The high conductivity observed in textile fibres coated with monolayer graphene and FLG make these materials valuable candidates for future applications requiring conductive textile fibres. Figure 5b compares the conductivity of our graphene-coated fibres with other types of conductive fibres reported in the literature taking into account the thickness of the conducting layer. The samples prepared in this work display considerably higher conductivity as compared to most of the fibres in the literature. These high values are due to the fact that conductivity is essentially two-dimensional, given the atomically thin nature of graphene films (further details in Supporting Information), whereas the other mentioned fibres have the conductive layer embedded in their structure. Furthermore, the conductivity of the fibres prepared by this method does not depend on the thickness of the coated fibre. Therefore, the outstanding electrical conductivity and optical transparency make graphene-coated fibres promising transparent conductors for future smart textile applications.

## Conclusions

We have demonstrated a versatile method to coat a wide range of textile fibres with graphene, including materials such as pure PP, PLA, PE and nylon. This method is applicable to tape-shaped fibres as well as cylindrical fibres. In addition to monolayer graphene, this method has the potential to transfer other types of graphene or 2D materials to textile fibres, as demonstrated by the FLG coating. Comparing the quality and continuity of the surface coverage of all the fibres, continuous graphene surface coverage is easily achieved without the need of a surface pre-treatment. Nevertheless, a pre-treatment of ultraviolet ozone can still improve the conductivity of the corresponding coated fibres, but often damaging them and making them more fragile. We have also demonstrated that, although the UVO pre-treatment of the fibres can lower their sheet resistance after the coating with graphene, it is possible to achieve low values with untreated fibres, especially if the surface is uniformly rough. In general, the sheet resistance of the fibres coated with graphene is lower for fibres produced with smoother surface, of the order of the kΩsq^−1^, and can withstand up to 1000 bending cycles down to a bending radius of 5 mm with no significant change in resistance. An extensive microscopic study of the surface of the coated fibres, by means of AFM, SEM and SThM, helped ruling out that the cracks often observed in conducting samples is caused by their manipulation or by significant height differences. The study also suggests that it is the stress to which the sheet is subjected during the transfer process that causes these small cracks, with minor effects on the sheet resistance. The same method to coat textile fibres with graphene was successfully employed to cylindrical nylon fibres, which were considerably smaller than the tape-shaped textile fibres. Sheet resistance values of the same order of magnitude were obtained, increasing with decreasing fibre diameter and good surface coverage was observed also without surface pre-treatment needed to achieve continuous coverage.

## Methods

Tape-shaped textile fibres were fabricated by Centexbel using a monofilament extrusion line from polypropylene (PP), polylactic acid (PLA) and polyethylene (PE). By varying the extrusion parameters, two types of PP fibres were produced: PP1 (0.04 mm thick, 1.6 mm wide); and PP2 (0.03 mm thick, 2.4 mm wide). The precursor was changed and compared PP and PE fibres with similar dimensions (0.03 mm thick, 2.4 mm wide). PLA-based fibres obtained from different grade precursors, *i.e.* PLA1 Bio-based polylactic acid polymer (0.1 mm thick x 1.2 mm wide) and PLA2 from PLA Naturework 6400D (0.07 mm thick, 1.0 mm wide) were produced. The fibres were rolled onto a bobbin and cut to the desired length. Commercial reels of monofilament nylon fishing lines were used: Mitchell® Marine Performance with 0.35 mm diameter (N1); Blue Pacific Expert Line Monofilament, 0.40 mm (N2); Climax® Monofilament Perlon, 0.55 mm (N3); Climax® Monofilament Perlon, 0.60 mm (N4).

### UVO Treatment

Ultra-Violet Ozone (UVO) treatment was performed using a UVO Cleaner® 144AX-220 (Jelight Company Inc.) for 11 minutes (6 minutes exposure time and 5 minutes exhaust time).

### Graphene Coating

The untreated fibres were coated with high quality monolayer graphene produced by cold-wall chemical vapour deposition^35^ and few-layer graphene (purchased from Graphene Supermarket) using the transfer method previously reported^23^.

### Profilometry

The surface profile parameters of the fibres were determined using a Taylor Hobson TalyScan 150 non-contact scanning instrument, at a speed of 30 µm/s and a spacing of 5 µm. To allow a direct comparison, PP1 and PP2 were placed side by side and measured in a single scan. A rectangular section with the same area was selected from each fibre to perform the calculations of the surface parameters. The same was done for PLA1 and PLA2, and using two sections of a PE fibre.

### Scanning Thermal Microscopy

All measurements were performed in ambient conditions using the PARK SYSTEMS XE7 Scanning Thermal Microscopy (SThM) equipment, with standard thermal probes from KELVIN Nanotechnology, thermoresistive probes where a Palladium resistive element is lithographically patterned on the AFM tip apex mounted on a Silicon nitride cantilever (400 nm thick). The electrical connection is made to two Au pads on the base of the probes. The thermal tip, with a radius of approximately 100 nm, has a sensitivity of ~1 Ω/°C and provides a 50 nm spatial resolution. The study was performed operating in the thermal conductivity contrast mode, with a bridge circuit to keep the probe at a constant temperature (determined by its resistance). During this type of measurement the tip is kept in contact with the sample. By electrically feeding the tip and monitoring the electrical current (output of the system in this mode) needed to keep its temperature constant during a scan, the thermal response of the material is detected in images of sub-micrometric resolution.

### Atomic Force Microscopy

AFM was performed with a Digital Instruments (Veeco/Bruker) Dimension 3100 with a Nanoscope IIIa controller in tapping mode using commercial Olympus Si tips (OTESP).

### Raman Spectroscopy

Raman spectra were measured in air and at room temperature with a Renishaw spectrometer using a 532 nm laser wavelength with a 1.5 μm spot size and a 1 mW of incident power.

### Electrical Measurements

Electric conductivity was measured by a two-probe method using tungsten probes and a Keithley 237 source-measure unit. The contacts were drawn with graphite paste to facilitate the measurements. Sheet resistance was calculated by subtracting the contact resistance and taking into account the aspect ratio of the graphene section on each sample. Contact resistance was estimated using a common method for graphene devices^36, 37^, based on measuring the two-probe resistance in devices with different contact separation while keeping the width of the channel constant. Since the two-probe resistance of a given sample is ${R}_{2p}={R}_{G}+2{R}_{C}$, where ${R}_{G}=\rho L/W$ is the graphene resistance ($\rho$ is graphene resistivity, $L$ is channel length and $W$ is channel width) and ${R}_{C}$ is the contact resistance, the contact resistance can be estimated from samples with different lengths ${L}_{1}$ and ${L}_{2}$ ($W$ is the same). In this case the contact resistance is given by ${R}_{2p}=({R}_{2p}^{Dev1}-{\rho }_{G}({L}^{Dev1}/{W}^{dev1}))/2$ and ${\rho }_{G}=({R}_{2p}^{Dev1}-{R}_{2p}^{Dev2}){({L}^{Dev1}/{W}^{Dev1}-{L}^{Dev2}/{W}^{Dev2})}^{-1}$. A contact resistance of the order of 1 kΩ was found for our devices, which is agreement with previous experiments^37^.

### Optical Measurements

A custom-built UV-Vis micro-spectrometer was used to acquire the transmission spectra. Light from an incandescent bulb was focused onto the samples through a condenser lens and collected by a X20 microscope objective, then delivered to the entrance slit of a spectrometer (Princeton Instruments Acton SP2500) equipped with a 1200 g/mm grating and CCD camera (Princeton Instruments PIXIS400). Transmittance as a function of wavelength λ was calculated as $T=I/{I}_{0}\,$where $I$ is the measured spectral intensity through the sample and ${I}_{0}$ is the incident light intensity. Corrections to account for the optics and gratings efficiencies at different wavelengths were applied using Princeton Instruments IntelliCal system.

### Scanning Electron Microscopy

Scanning electron microscopy (SEM) was performed using a Hitachi SU-70 scanning electron microscope at an accelerating voltage of 4 kV, working distance of 6200 µm, emission current of 44000 nA and magnifications of 100x, 200x, 500x, 1000x and 10000x.

## Electronic supplementary material

Supplementary Information
