# Supplementary material for: Towards conductive textiles: coating polymeric fibres with graphene
Source: Sci Rep. 2017 Jun 26;7:4250. doi: 10.1038/s41598-017-04453-7 (PMC5484677; doi:10.1038/s41598-017-04453-7)
Supplement: Supplementary file 1 — Supplementary Information [file 41598_2017_4453_MOESM1_ESM.pdf]

Supplementary Information

Towards conductive textiles: coating polymeric fibres with graphene

Ana I. S. Neves[1,2,*], Daniela P. Rodrigues[3], A. De Sanctis[1], Elias Torres Alonso[1], Maria S. Pereira[3], Vitor S. Amaral[3], Luis V. Melo[2,4], Saverio Russo,[1] Isabel de Schrijver[5], Helena Alves[3,4] Monica F. Craciun[1]

[*]a.neves@exeter.ac.uk

[1] Centre for Graphene Science, College of Engineering, Mathematics and Physical Sciences, University of Exeter, EX4 4QF Exeter, United Kingdom

[2] INESC-MN and IN, Rua Alves Redol 9, 1000-029 Lisbon, Portugal

[3] Department of Physics and CICECO, University of Aveiro, 3810-130 Aveiro, Portugal

[4] Department of Physics, Instituto Superior Técnico, University of Lisbon, Av. Rovisco Pais 1, 1040-001 Lisbon, Portugal

[5] CenTexBel, Technologiepark-Zwijnaarde 7, 9052 Ghent, Belgium

a

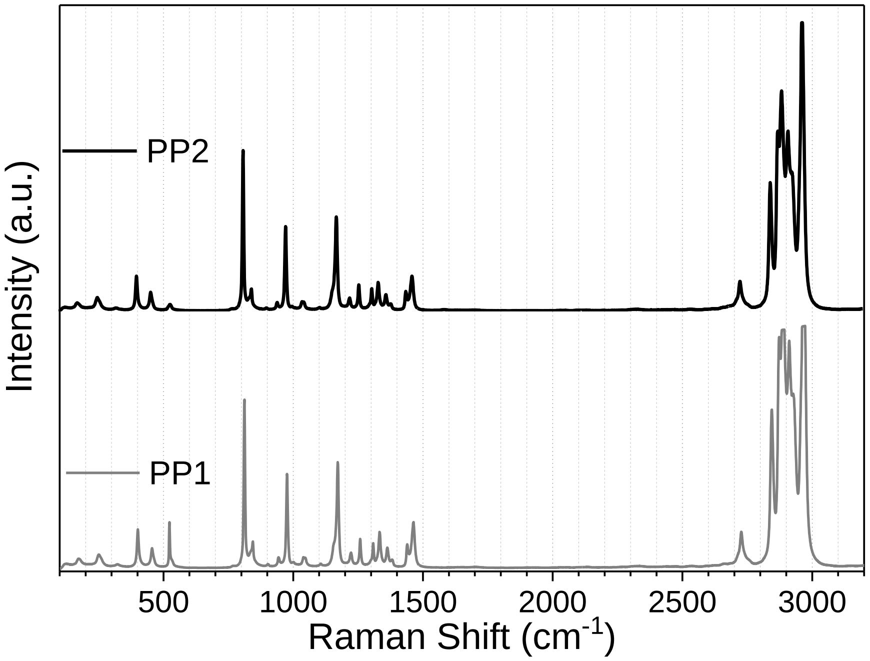

b

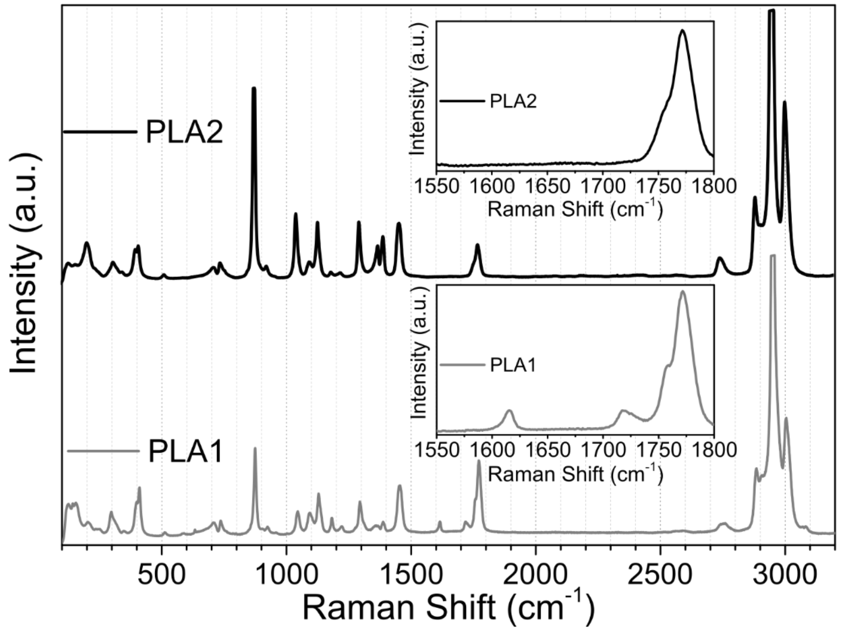

Figure S1. Full Raman spectra of **(a)** PP1 (grey, bottom) and PP2 (black, top); and **(b)** PLA1 (grey, bottom) and PLA2 (black, top). Insets show in detail the two additional peaks in PLA1 at 1615 cm^{-1} and 1717 cm^{-1}.

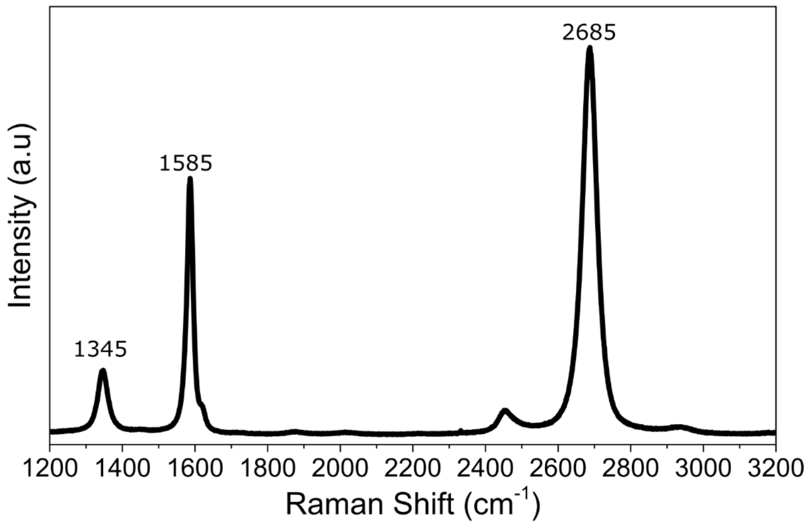

Figure S2. Raman spectra of the same type of graphene used to coat the fibres transferred to SiO_2, with main peaks D, G and 2D, centred at 1345, 1585 and 2685 cm^{-1}, respectively.

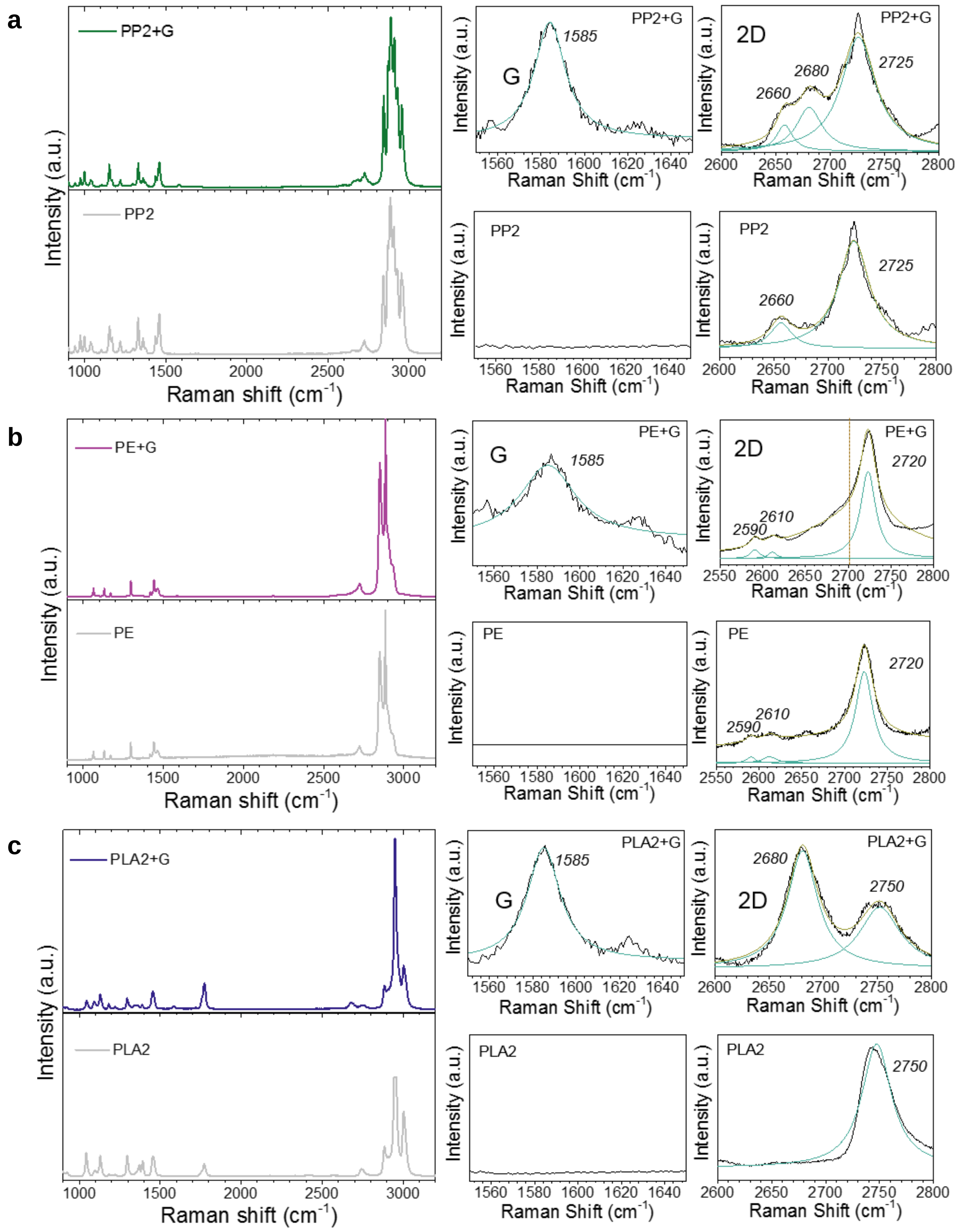

Figure S3. Extended Raman spectra for the fibres with and without graphene (left) and details of the G and 2D peak regions (right) with corresponding integrated peaks for: **(a)** PP2; **(b)** PE and **(c)** PLA2.

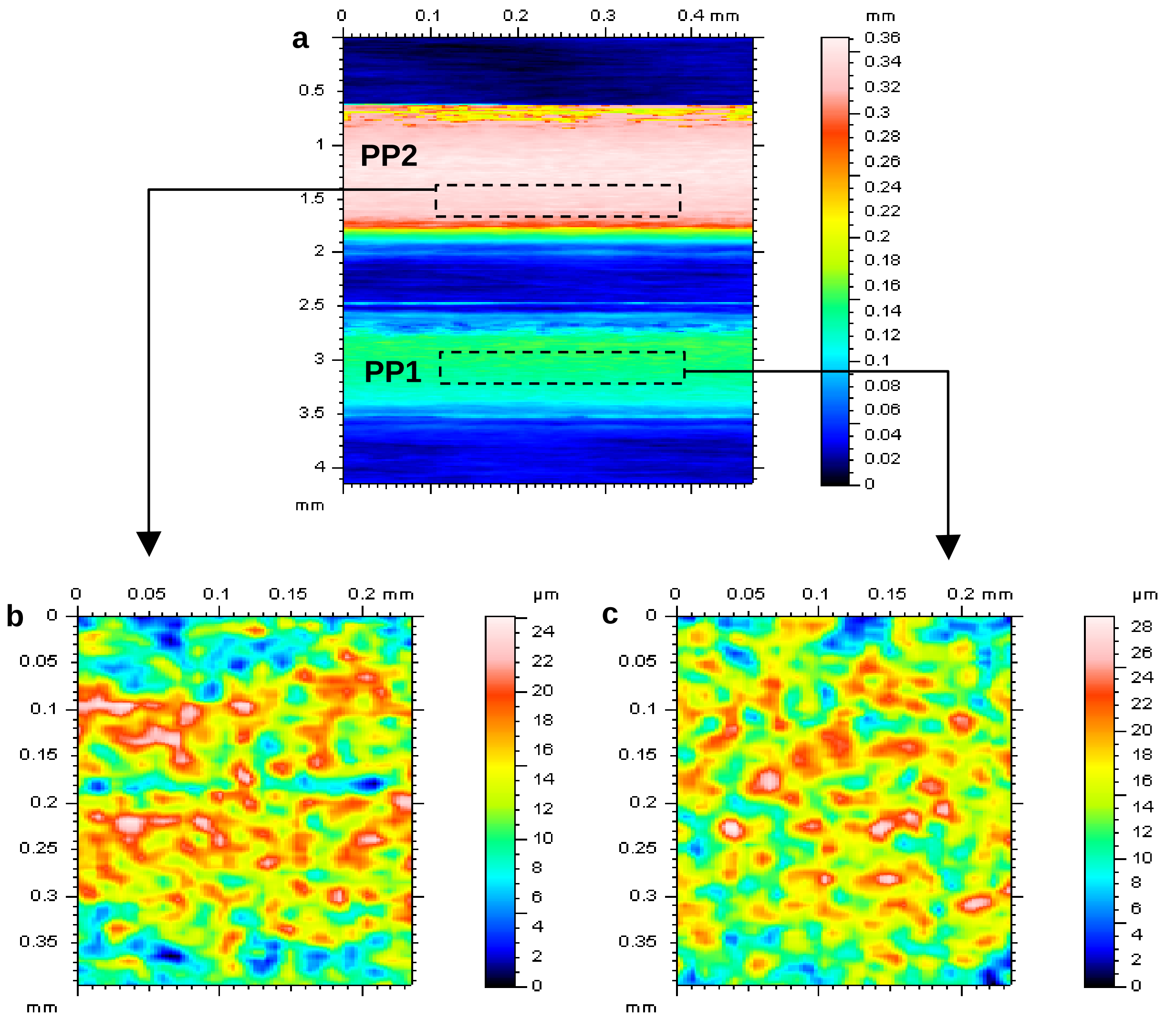

Figure S4. Height profiles determined using a non-contact scanning instrument: **(a)** PP2 and PP1 in the same scanning area, with rectangles illustrating where areas of 235 x 395 were taken for PP2 **(b)** and PP1 **(c).**

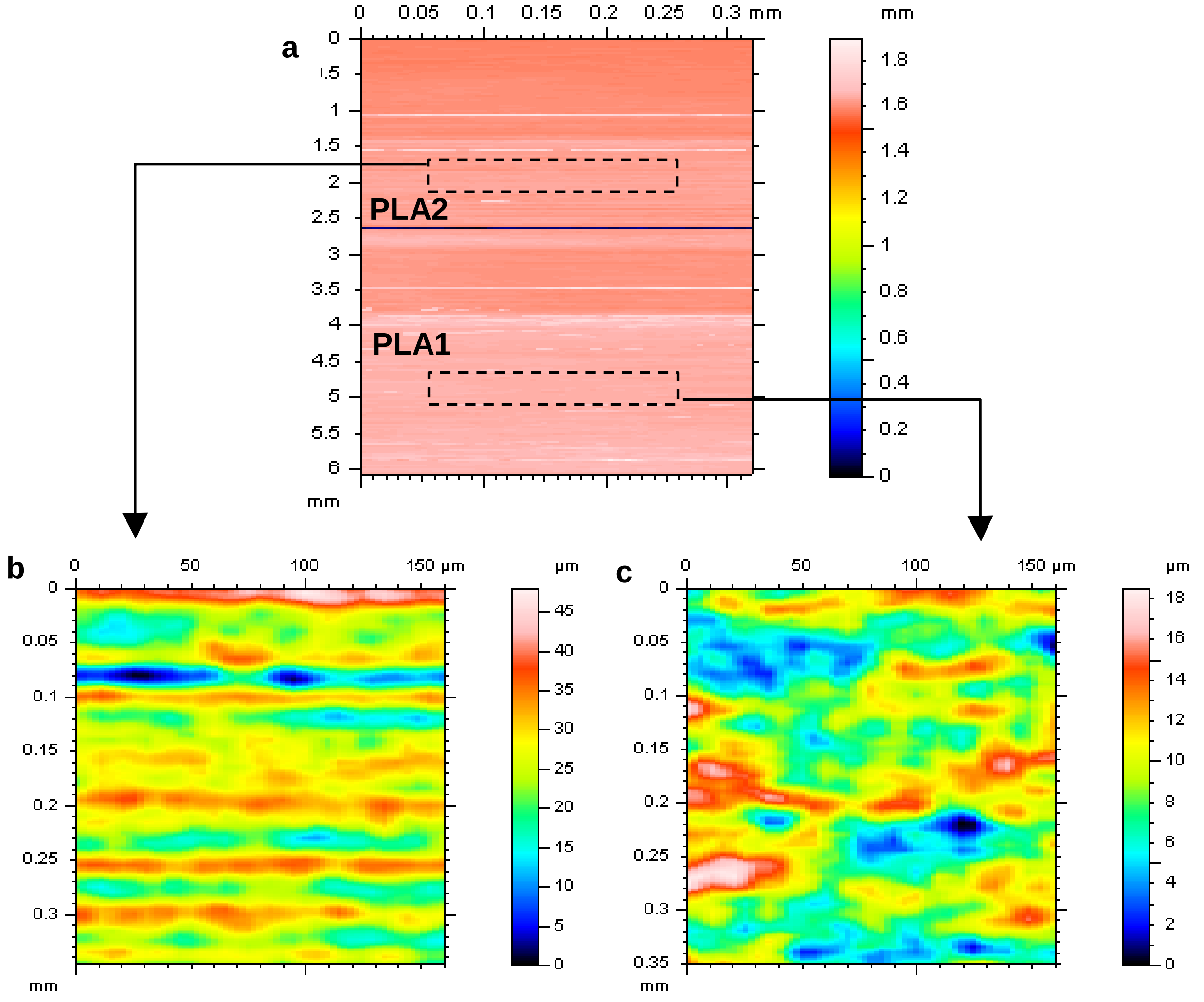

Figure S5. Height profiles determined using a non-contact scanning instrument: **(a)** PLA 2 and PLA1 in the same scanning area, with rectangles illustrating where areas of 160 x 360 were taken for PLA2 **(b)** and PLA1 **(c)**.

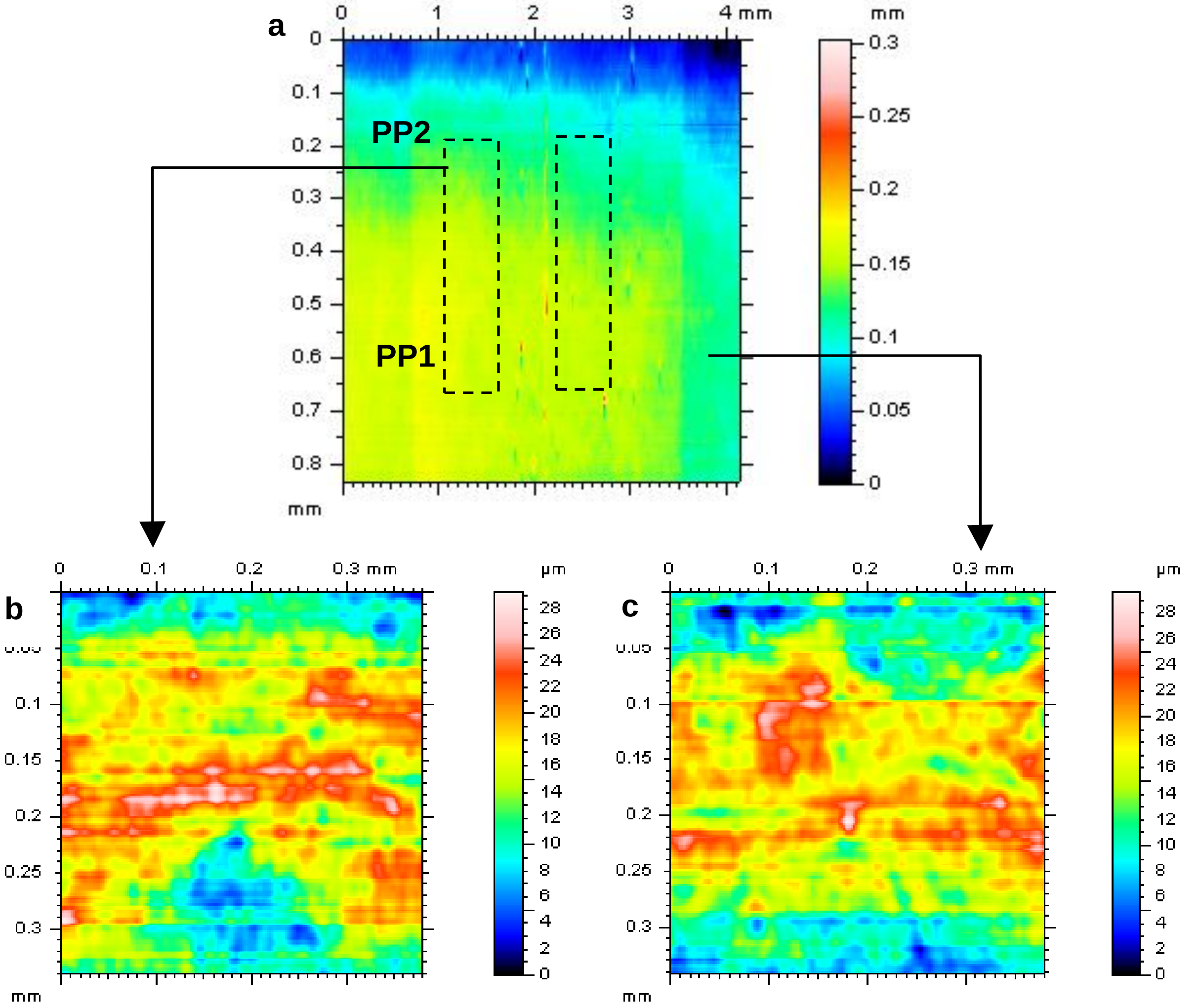

Figure S6. Height profiles determined using a non-contact scanning instrument for two sides of the same PE sample: **(a)** scanning area showing the whole sample, with rectangles illustrating where areas of 415 x 840 were taken for PEa, left hand side **(b)** and PEb, right hand side **(c).**

Table S1. Surface Parameters for PLA1, PLA2, PP1, PP2 and two sizes of the same PE sample, PEa and PEb, calculated from Figures S1b, S1c, S2b, S2c, S3b and S3c, respectively. The width and length of the scanned area for the calculation of the parameters is also listed.

Parameter	PLA1	PLA2	PP1	PP2	PEa	PEb
Sa (µm)	2.4	5	3.27	3.4	3.95	4
Sq (µm)	2.96	6.46	4.09	4.16	4.82	4.77
Sp (µm)	9.55	22.3	14.1	12.1	13.6	14.5
Sv (µm)	8.96	25.7	14.8	13.1	15.7	15.1
St (µm)	18.5	48	28.9	25.1	29.3	29.6
Ssk	0.201	-0.303	0.00236	-0.00773	-0.186	-0.139
Sku	2.83	3.83	3.01	2.53	2.46	2.3
Sz (µm)	18.3	48	23.2	25.1	24.3	24.2
Width (µm)	160	160	235	235	415	415
Length (µm)	350	350	395	395	840	840

Sa: Average Roughness
Sq: Root Mean Square Roughness
Sp: Maximum Peak Height
Sv: Maximum Valley Depth
St: Total Height
Ssk: Skewness
Sku: Kurtosis
Sz: Maximum Height of Surface

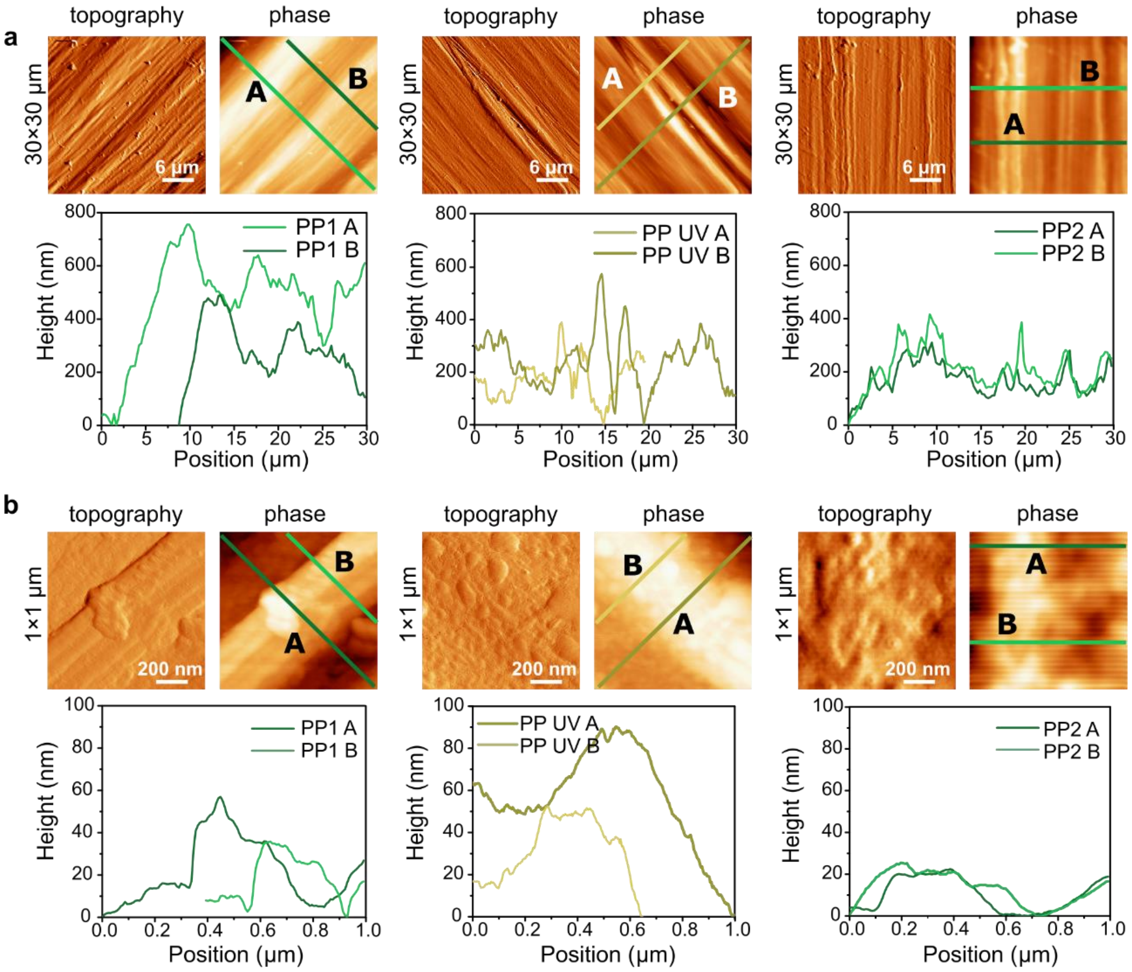

Figure S7: AFM amplitude and topography 30×30 µm (**a**) and 1×1 µm (**b**) images of the PP fibres before coating: first batch (PP1, left); UVO-treated first batch (PP1 UV, middle); second batch (PP2, right) and corresponding height profiles of transverse lines with respect to the extrusion axis (bottom).

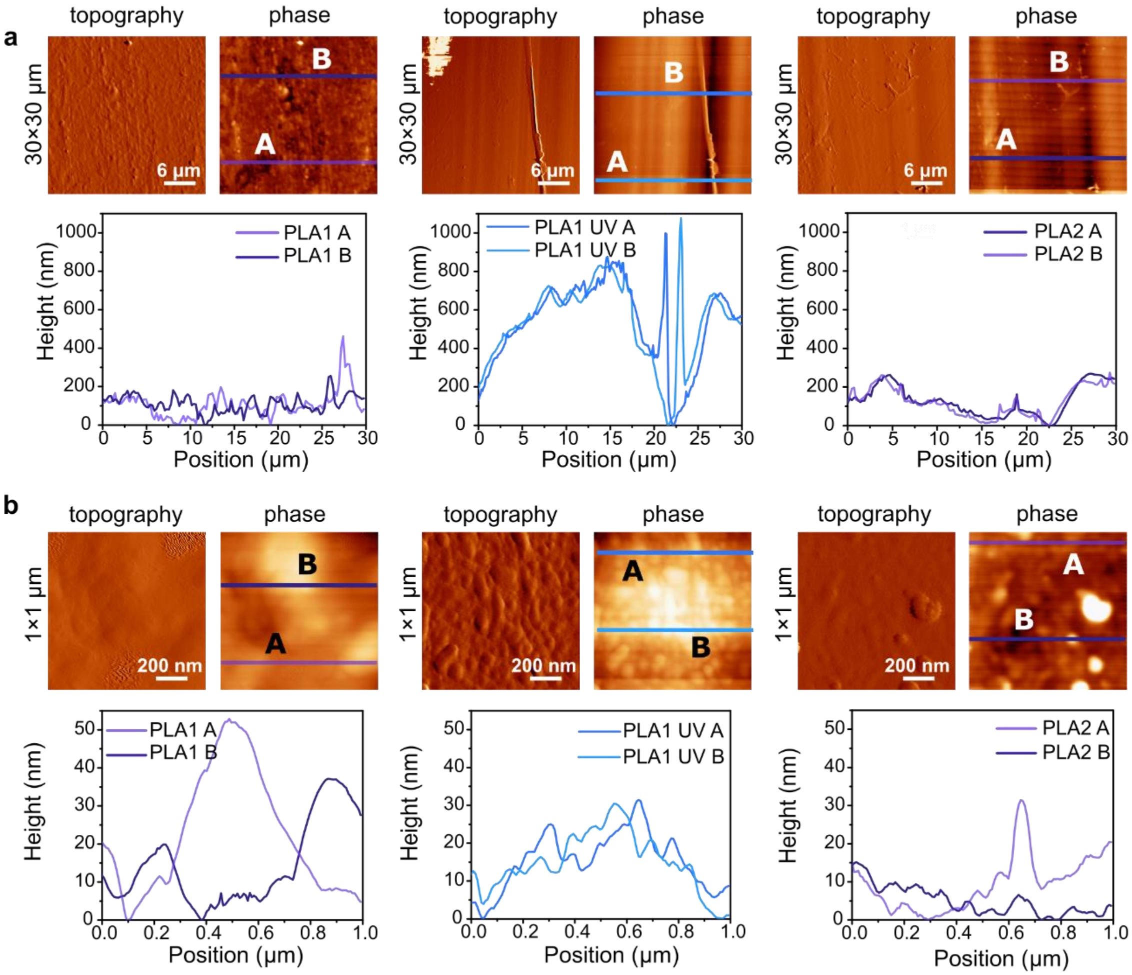

Figure S8: AFM topography and phase 30×30 µm (**a**), and 1×1 µm (**b**) images of the PLA fibres before coating: first batch (PLA1, left); UVO treated first batch (PLA1 UV, middle); second batch (PLA2, right) and corresponding height profiles of transverse lines with respect to the extrusion axis (bottom).

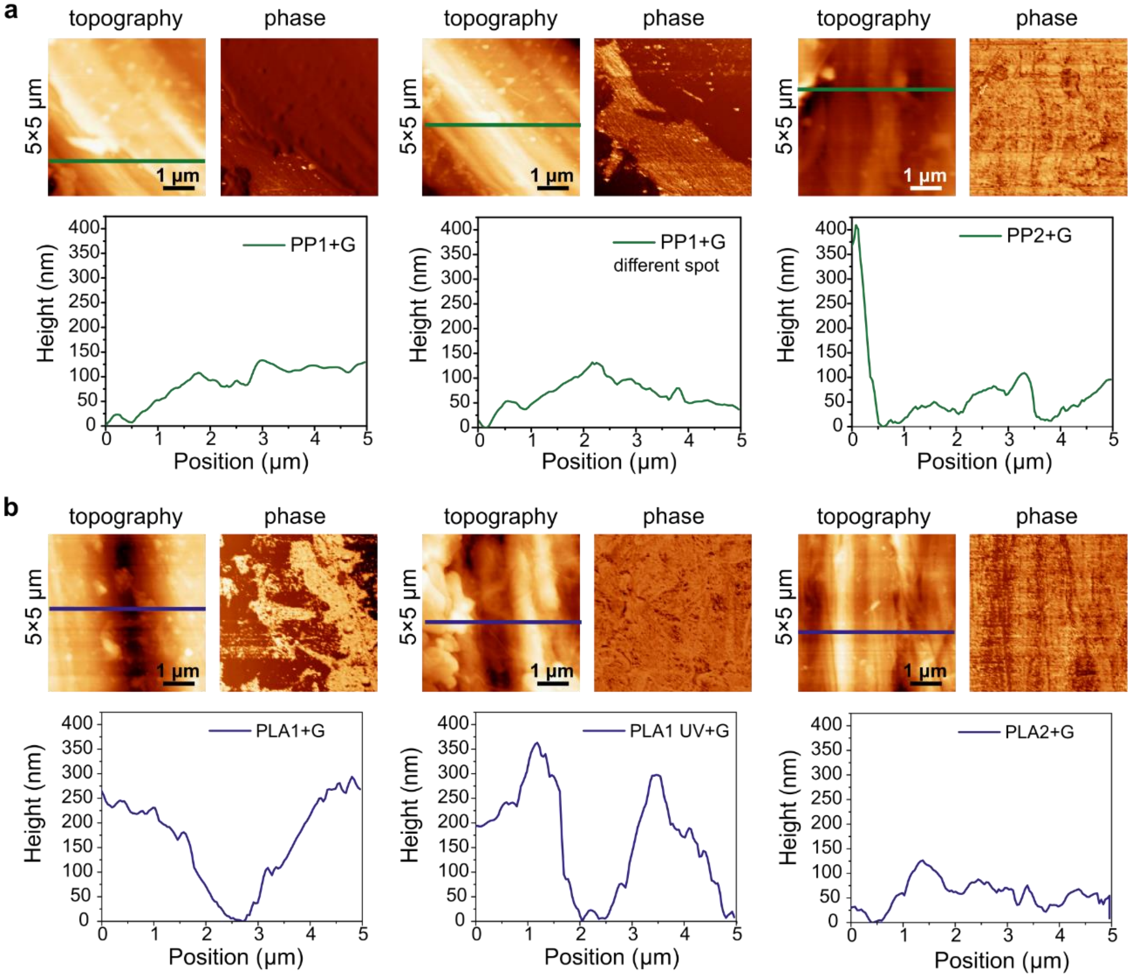

Figure S9: AFM topography and phase 5×5 µm images of graphene-coated (**a**) PP1 (2 different spots) and PP2 and (**b**) PLA1; PLA1 UV and PLA2 (bottom).

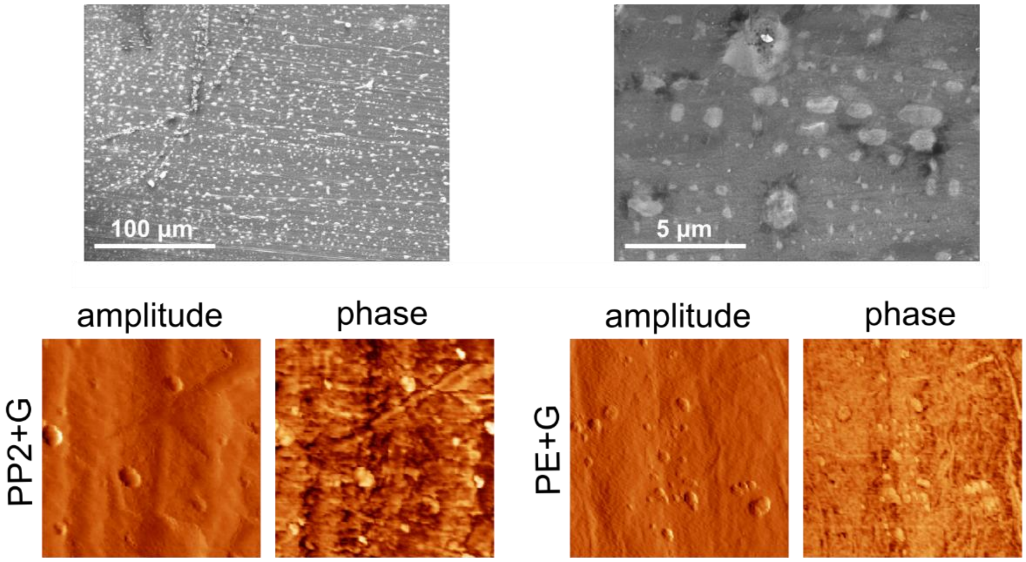

Figure S10. PMMA residues visible in SEM images of graphene-coated PP1 fibres (top), and in AFM images of PP2 and PE fibres (bottom).

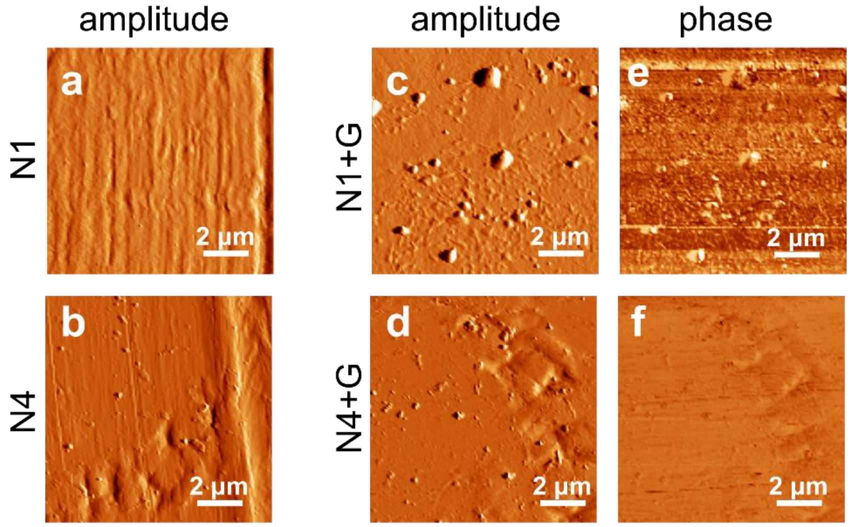

Figure S11. AFM amplitude images of uncoated N1 (**a**) and N4 (**b**) nylon fibres; AFM amplitude and phase images of graphene-coated N1 (**c** and **e**) and N4 (**d** and **f**) nylon fibres.

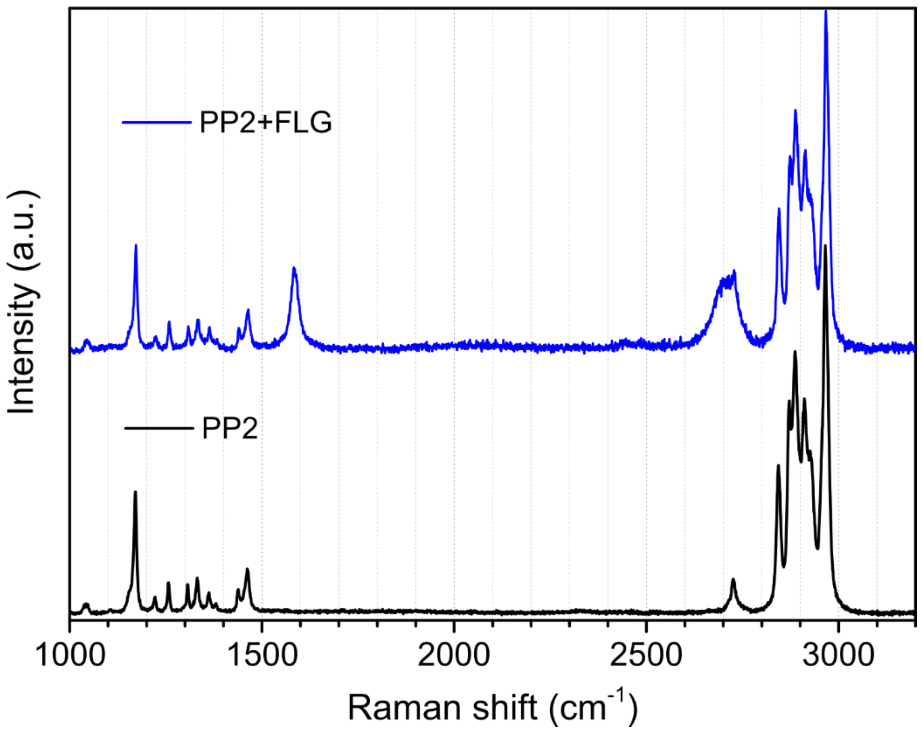

Figure S12. Raman spectrum of bare PP2 fibre (black) and PP2 coated with few-layer graphene (blue).

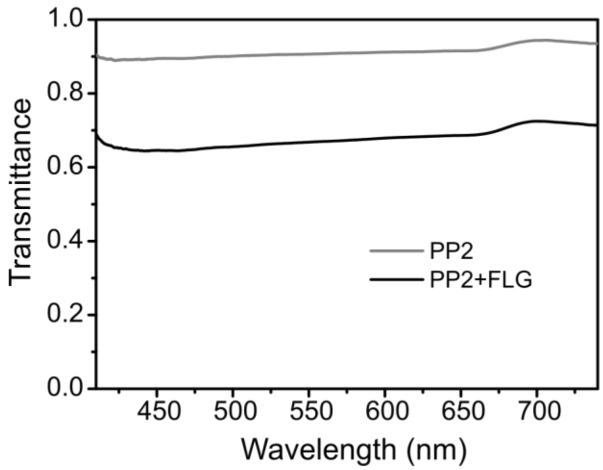

Figure S13. Transmittance as a function of wavelength for bare PP2 fibre (grey) and PP2 coated with few-layer graphene (black).

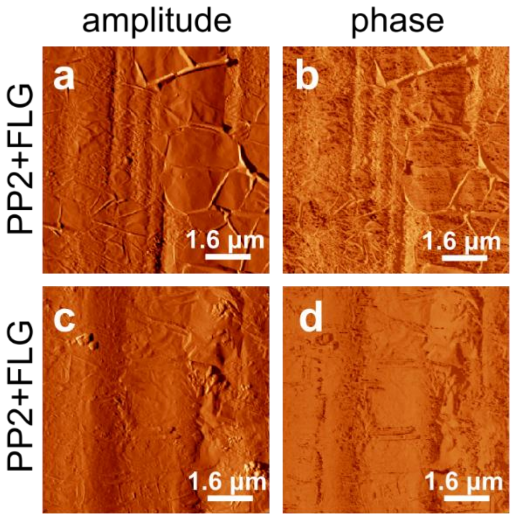

Figure S14. AFM amplitude and phase images of two different PP2 fibres covered with few-layer graphene (FLG), with **a** and **b** showing portions of the upper graphene that appeared to be cracked and lifter towards the edges, and **c** and **d** showing a smoother and more homogeneous surface.
